# Tensions and Paradoxes of Stigma: Discussing Stigma in Mental Health Rehabilitation

**DOI:** 10.3390/ijerph17165943

**Published:** 2020-08-16

**Authors:** Jenny Paananen, Camilla Lindholm, Melisa Stevanovic, Elina Weiste

**Affiliations:** 1 Department of Finno-Ugrian and Scandinavian Studies, Faculty of Arts, University of Helsinki, P.O. Box 54, 00014 Helsinki, Finland; Elina.Weiste@helsinki.fi; 2 Faculty of Information Technology and Communication Sciences, Tampere University, 33014 Tampere, Finland; Camilla.Lindholm@tuni.fi; 3 Faculty of Social Sciences, University of Helsinki, 00014 Helsinki, Finland; Melisa.Stevanovic@helsinki.fi

**Keywords:** content analysis, discrimination, focus group, interview, mental health, prejudice, psychosocial health, rehabilitation, stigma, qualitative study

## Abstract

Mental illness remains as one of the most stigmatizing conditions in contemporary western societies. This study sheds light on how mental health professionals and rehabilitants perceive stigmatization. The qualitative study is based on stimulated focus group interviews conducted in five Finnish mental health rehabilitation centers that follow the Clubhouse model. The findings were analyzed through inductive content analysis. Both the mental health rehabilitants and the professionals perceived stigmatization as a phenomenon that concerns the majority of rehabilitants. However, whereas the professionals viewed stigma as something that is inflicted upon the mentally ill from the outside, the rehabilitants perceived stigma as something that the mentally ill themselves can influence by advancing their own confidence, shame management, and recovery. Improvements in treatment, along with media coverage, were seen as the factors that reduce stigmatization, but the same conceptualization did not hold for serious mental illnesses. As the average Clubhouse client was thought to be a person with serious mental illness, the rehabilitation context designed to normalize attitudes toward mental health problems was paradoxically perceived to enforce the concept of inevitable stigma. Therefore, it is important for professionals in rehabilitation communities to be reflexively aware of these tensions when supporting the rehabilitants.

## 1. Introduction

The people living today belong to the same species, but their various group memberships are a constant source of inequality, prejudice, injustice, and discrimination. Certain ethnic groups may be perceived negatively in the moral and ethical domains, working-class people in the domain of intelligence and competence, women in the technical and mathematical domain, and so on. Some groups, such as those who are mentally ill, suffer from a more severe global devaluation. Such devaluation encompasses not only individual experience but is also part of a wider scope of societal development, historical contingencies, power relations, community practices, and policies.

Stigma is the central notion pertaining to people’s tendency to devaluate others. It is a concept that refers to “a mark of shame or discredit” (Merriam-Webster Dictionary) that leads others to disapprove of or hold negative beliefs about certain individuals, qualities, or circumstances. The most influential theoretician of the 20th century to provide a theory of stigma was Erving Goffman (1963). Goffman’s stigma theory draws on the notion of stigma as a process, whereby the reactions of others spoil normal identity. In this process, a person with an attribute that is discredited by their society is rejected because of this attribute [1].

In this paper, we discuss how mental health rehabilitants and the professionals working with them perceive the devaluations, such as stigma, that are associated with mental illness. First, we will provide an overview of the literature, focusing on how stigma is manifested in mental illnesses.

## 2. Mental Illness and Stigma

The stigmatization of people with mental illness demonstrates a millennia-long history of social exclusion and prejudice, connecting mental disorders to the belief in the Middle Ages that they are a punishment from God and to genocide during the Nazi reign in Germany [2]. As pointed out by Stier and Hinshaw [3], stigma against mental illness has persisted even though tolerance for other stigmatized groups has increased. In a survey that included respondents from 229 countries [4], up to 16% of the respondents believed people with mental illness to be more violent than others. Further, these public perceptions are often endorsed by negative media portraits of mental illnesses, focusing on topics such as crimes, violence, and suicide, and people with mental illness being perceived as a threat to others [5,6]. The stigmatization of a person with mental illness also seems to be influenced by the respondent’s culture; more respondents in developing countries connected mental illness with violence than respondents in developed countries did, see also [7]. In the Finnish context, Aromaa used a population survey to investigate the stigmatization of people with mental disorders [8]. Aromaa’s results indicate that the majority of respondents did not consider people with depression to be responsible for their illness. However, most of the respondents believed that the recovery of people with depression was their own achievement.

Stigma affects persons with mental illness in multiple ways [9]. They are challenged both by their health issues and by others’ attitudes and prejudices toward their illness [10]. The notion of self-stigma refers to an individual’s negative beliefs about the self, caused by the process of an individual with mental illness internalizing the stigmatizing ideas of society [10,11,12,13] (for self-stigma and concealable minorities, see [14]). Internalized stigma can manifest, for example, as the so-called “why try” effect [15]. This effect can be defined as individuals with mental illness applying the existing stereotypes to themselves, thus believing that they are incapable of achieving personal goals, such as finding a workplace or making friends [15]. Stigma can also affect those who are closely associated with stigmatized individuals, and this is known as “courtesy stigma”, see [1]. This affects both families of people with mental illness [16,17,18,19,20,21,22]—for an interesting parallel, see [23] on affiliate stigma in parents of children with Autism Spectrum Disorder, and mental health professionals [24,25,26,27,28].

There is a substantial body of research on how stigma impedes mental health rehabilitation. In a seminal paper from 2004, Corrigan outlines how stigma both decreases the self-esteem of people with mental health problems and robs these people of social opportunities, in this manner impeding these people from seeking mental health services [18,29]. Apart from affecting the help-seeking behavior of people with mental illness, research has identified a connection between stigma and adherence to psychosocial treatment in people with mental illness. Fung, Tsang, and Corrigan [30] noted that higher levels of self-stigma were associated with poor participation in psychosocial treatment, whereas better self-esteem was a significant indicator of better attendance at psychosocial treatment. Further, self-stigma can have a negative impact on recovery [31]. Therefore, it is an interesting empirical question how individuals with mental illness perceive, explain, and account for mental illness stigma. Is it something that is unfairly inflicted upon the individuals by society or something that the individuals themselves are at least partially responsible for?

Stigma does not affect only people with mental health problems, but also health care professionals. For example, previous research has identified the phenomenon of diagnostic overshadowing, which refers to the process in which physical symptoms are misattributed to mental illness. This phenomenon is assumed to relate to premature mortality in people with mental illness see, e.g., [32]. There is also evidence that health care providers’ treatment decisions can be influenced by the endorsement of stigma about mental illness. For example, care providers who agree with stigmatizing characteristics have been shown to be more pessimistic about mental health patients’ adherence to treatment, which, in turn, can affect their willingness to refer the patients to specialists and prescribe medicine [33].

When it comes to mental health professionals, one would expect that their attitudes toward people with mental illness would be better compared to the general population since their profession involves dealing with mental illness as a normal part of life. However, previous research has demonstrated that even mental health professionals may hold negative beliefs about individuals with mental illness and contribute to their discrimination. For example, a survey conducted by Nordt, Rössler, and Lauber [34] showed that psychiatrists had more negative stereotypes of people with mental illness than the general population. Further, their study demonstrated that mental health professionals wanted to keep a large social distance from people with mental illness, see also [35]. The results by Loch and colleagues [36] even indicated that compared to the general population, psychiatrists held stronger negative stereotypes of individuals with schizophrenia. According to Rössler [2], these negative attitudes of mental health professionals can most likely be attributed to the fact that they frequently encounter patients who are noncompliant to treatment. This tension between the overall expectation of understanding and empathetic attitudes of mental health professionals toward their patients’ conditions, on one hand, and the less ideal reality, on the other hand, is something that professionals may feel uneasy about.

In this paper, our aim is to shed light on some inherent tensions and paradoxes of stigma in the context of community-based mental health rehabilitation. We investigate the following: (1) How mental health rehabilitants and professionals perceive, explain, and account for mental illness stigma; (2) How the participants’ stigma talk is associated with their institutional positions as professionals or service users.

We assume that the answers to these questions will increase the overall understanding of stigma in contemporary mental health rehabilitation, ultimately helping us unveil the possible tensions in the views of mental health rehabilitants and professionals.

## 3. Materials and Methods

Our data consist of 10 video-recorded focus group interviews that were organized in five Finnish Clubhouses. The Clubhouse model is a worldwide concept involving local community centers that offer people with mental illnesses a sense of belonging, opportunities for social relationships, and support in obtaining employment, education, and housing [37]. A Clubhouse is a membership organization, which means that it is open to anyone with a history of a mental disorder, and that the people who participate in the activities at the Clubhouse are considered its members [37]. In this study, however, we refer to the Clubhouse members as “clients” or “mental health rehabilitants” to avoid confusion with the term “staff member”, which refers to the support workers. Nevertheless, the idea of membership is important to the Clubhouse ideology, as the people with mental illness are treated not as patients with a certain diagnostic label but as co-participants or colleagues who have something to contribute to the joint aims of the organization. A referral is not needed, and the clients are not expected to disclose their diagnoses or illness histories unless they wish to do so. The role of the paid staff is to work side by side with the clients in all functions of the house, engage with them, and encourage them to reach their full potential [37]. One aim of the Clubhouses is to reduce the stigma surrounding mental illnesses [38]. This is achieved, for instance, by reducing unemployment among clients with a Clubhouse-owned Transitional Employment program [38] and launching anti-stigma campaigns (such as a “Diagnosis-free zone”) on social media. 

The Clubhouse units were chosen to be included in the study through a purposive sampling designed to provide maximum variation. The chosen units varied in age, geographical location, and number of members. The interview tour continued until the saturation point was reached. Two groups in each house were interviewed: one group consisted of clients and the other comprised support workers. To promote inclusion, participants were recruited through an open call and not selected based on any particular criteria. All voluntary participants were accepted. There were between three and seven participants in each group, as well as one interviewer, who conducted all of the interviews. All of the participants were adults who had been involved in a Clubhouse community from 1 to 21 years.

The structure was similar in each interview, but the groups were encouraged to continue the discussion freely around each question (for more details on focus groups as a method, see [39,40]). The main theme of the interview was everyday life and interaction at the Clubhouse. The duration of the interviews varied between 73- and 113-min. Stigma was discussed late in the interview in order to give the group time to adjust to the interview situation before moving on to this particularly sensitive topic.

Focus groups are suitable for studying comprehension on known issues, such as stigma related to mental health issues, cf., [41]. The method provides the participants a platform for discussing sensitive themes from their viewpoints and on their own terms; the questions are not targeted at individuals, but the participants are encouraged to talk to one another [42]. As the method allows thinking together and comparing and adjusting views within the group, it provides evidence about the societal understandings and opinions that people are ready to support in public. Group discussions can also facilitate self-disclosure, as people may feel more supported in group situations with their peers than they would on their own [43]. 

In this paper, we examined the stimulated discussions on the stigma related to mental health problems. As stimulation, the groups were given three cards with percentages printed on them: 47%, 62%, and 71%. They were told that one of the answers depicted the percentage of mental health rehabilitants that experience stigmatization because of their mental illness in Finland, according to the Finnish Mental Health Barometer [44]. For the barometer, a total of 300 mental health rehabilitants and their family members were interviewed via telephone. The informants were selected randomly from the membership register of the National Congress of Mental Health. In addition, the barometer utilized an internet survey for mental health professionals (N = 624). 

In the interview, the groups were asked to work together and choose the card they thought correctly depicted the prevalence and to provide explanations for their choice. After their decision, the interviewer revealed the result of the barometer (47%) and asked the group to deliberate. Thus, the stimulated group discussion consisted of the following stages:

1. The interviewer introduces the topic and the cards.
2. The group contemplates which card to choose.
3. The group justifies their choice.
4. The interviewer reveals the result of the national barometer.
5. Broad discussion.
6. The interviewer shifts the topic.

The interviewer’s task was to encourage the groups to think aloud and discuss the theme broadly. If the groups’ own evaluation differed from the barometer’s result, the groups were asked to contemplate the reasons for the divergence. Before the interview, the groups were also encouraged to provide examples and anecdotes whenever they found fitting. The interviewer refrained from leading the group to any conclusions and moved on to the next stage only after the discussion seemed to cease or drift too far from the topic.

The interviews were transcribed for the study and analyzed using the qualitative method of inductive content analysis [45]. The content of the interviews was systematically labeled using the data analysis software NVivo12. First, the topics around the theme of stigmatization were coded and categorized, and then recurrent patterns and connections between categories were identified. We present the visualizations of our content analysis beside our results.

The study was conducted in accordance to the Declaration of Helsinki and the project acquired a statement of validity from the Ethical Review Board in the Humanities and Social and Behavioural Sciences in the University of Helsinki (17 December 2019, 69/2019). All participants were given written and oral information about the study and the data management, and each participant signed a written consent. As the identities of the participants are confidential, all personal data were omitted or changed during the transcription process. In addition to the recorded interviews, no other information was collected from the participants. In the extracts, the participants were identified with pseudonyms.

## 4. Results

Our results show how the mental health professionals and rehabilitants evaluated and rationalized the prevalence of stigma (Section 4.1) and how they justified the divergence between their evaluation and the barometer’s result (Section 4.2). The analysis revealed two main themes. On one hand, the groups appealed to current trends and ongoing societal changes (Section 4.2.1), and on the other, they underlined their familiarity with the rehabilitation context and their particular perspective as rehabilitation clients or professionals (Section 4.2.2). The differences between the conceptions of the mental health rehabilitants and the support workers are addressed in the following. As many of the themes examined in this paper have been studied previously from another angle, we will compare our findings with those described in previous literature throughout the analysis.

### 4.1. Evaluation Task

In the stimulation task, in which the groups were asked to evaluate the prevalence of experiences of stigmatization among mental health rehabilitants, none of the groups chose the card that depicted the percentage presented in the Finnish mental health barometer, namely, 47% [44]. Instead, all five support worker groups chose the highest percentage presented on the three cards: 71%. Out of the client groups, three chose 71% and two decided on 62%. Hence, all of the groups believed that the majority of mental health rehabilitants experience stigmatization in modern day Finland, and the professionals’ view was even more pessimistic than the clients’.

**Figure 1:**
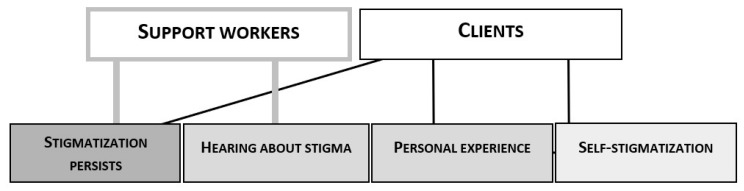
Visualization of the support workers’ and clients’ most common explanations for their choice in the stimulation task (categories with three or fewer instances are not depicted).

The participants’ common explanations for their decision in the stimulation task are illustrated in Figure 1. As shown in the figure, both the support workers and the clients used the persistence of stigma as an argument for believing in its widespread prevalence. They all acknowledged stigmatization as a problematic and serious phenomenon.

By depicting the prevalence as still strong, the participants indicate that stigma has also existed in the past (Extracts 1–3). The underlying idea seems to be that there have been efforts to diminish stigma, but they have not made a notable difference (Ext. 4). The participants’ notions of the past seem to refer to the historical burden related to the treatment of the mentally ill in western societies, cf. [2], but the topic was explicitly talked about only later in the interview (we come back to this issue in Section 4.2.1).

1. Anja, support worker: “I think it is still like this” (points at 71%).
2. Seija, support worker: “It’s a high rate anyway. Could it still be at 71%?”
3. Jonna, client: “Stigma is (--) still extremely strong.”
4. Oiva, client: “The situation with stigmatization has not improved much.”

The support workers also appealed to having often heard about incidents of discrimination. They explained that the clients often discuss experiences of stigmatization at the Clubhouse. What is noteworthy is that the support workers in different houses produced very similar narratives of mental health rehabilitants not being heard in primary care consultations because of their diagnosis (Ext. 5–7).

5. Anja, support worker: “One can be Otherwise ill or in pain, but it is always automatically linked to… Oh you have these psychological issues so these are probably just symptoms of that then.”
6. Ulla, support worker: “Yeah, and if you go see a doctor or something and you have an entry, whether it is about mental health, addiction or anything, you will definitely be treated differently.”
7. Maikki, support worker: “I’m thinking of the people who have a physical condition. When they go see someone in primary care, like, what does it take for them to be heard? Like, when you think they have a mental health problem.”

In the foregoing, we have mentioned how diagnostic overshadowing can increase the risk of health complications for people with mental illness, see also [32]. As demonstrated in Extracts 5–7, our study shows not only that the experiences of diagnostic overshadowing are shared in ordinary conversations in mental health rehabilitation communities, but also that these narratives may form a basis for the general understanding of stigma as a phenomenon.

The clients, on the other hand, appealed to their own first-hand experiences rather than others’ reports. Some confessed that they had lost friends and other social contacts due to their illness. However, incidents of diagnostic overshadowing were not mentioned, which suggests that from the clients’ perspective, the attitudes of their close contacts are central with regard to their experience of belonging. Some groups also talked about misconceptions they have encountered, such as the belief that mental illnesses are a matter of attitude and not actual illnesses (Ext. 8).

8. Mari, client: “Older people, in particular, think that mental health problems are not health problems.”
  Mikko, client: “Self-inflicted.”
  Mari: “We could get rid of them just by deciding (*laughs*) not to be bipolar or whatnot anymore.”

The clients were also aware of self-stigmatization and its effect on how they perceive other people’s attitudes towards themselves (Ext. 9–11) [10,11,12,13]. Some even implied that the experiences of marginalization can arise from self-shame that is projected onto others rather than inflicted by others (11).

9. Milla, client: “When you become ill, the shame is a heavy burden. One thinks, I am so shameful that everyone else will condemn me as well.”
10. Saku, client: “It is a very personal experience. Do you feel like you are being labeled? Do you see yourself as different in some way?”
11. Mari, client: “I possibly see it that way. That many people experience stigmatization although others do not stigmatize them that much.”

The clients also agreed that acknowledging one’s own problems and their consequences is key to accepting one’s self and conquering the shame (Ext. 12). This notion is in line with previous studies. For example, being comfortable with using mental health services correlates with having fewer discriminating beliefs about mental illnesses [33], whereas the fear of stigma decreases help-seeking initiatives [46,47].

12. Netta, client: “When one gets back on one’s feet, one sort of recovers. Like, I have this thing but maybe it’s okay. It then decreases, and one starts to think that it isn’t that terrible to have something.”

What is interesting is that the trained support workers viewed stigmatization as something that is forced on the mentally ill, whereas the clients’ discussions left more room for the rehabilitants’ own agency. That is, having confidence in one’s self can reduce the experiences of stigma. We will investigate the viewpoints of the clients and the professionals further in the next section, in which we analyze how the participants received the result of the national barometer.

### 4.2. Accounting for the View

**Figure 2:**
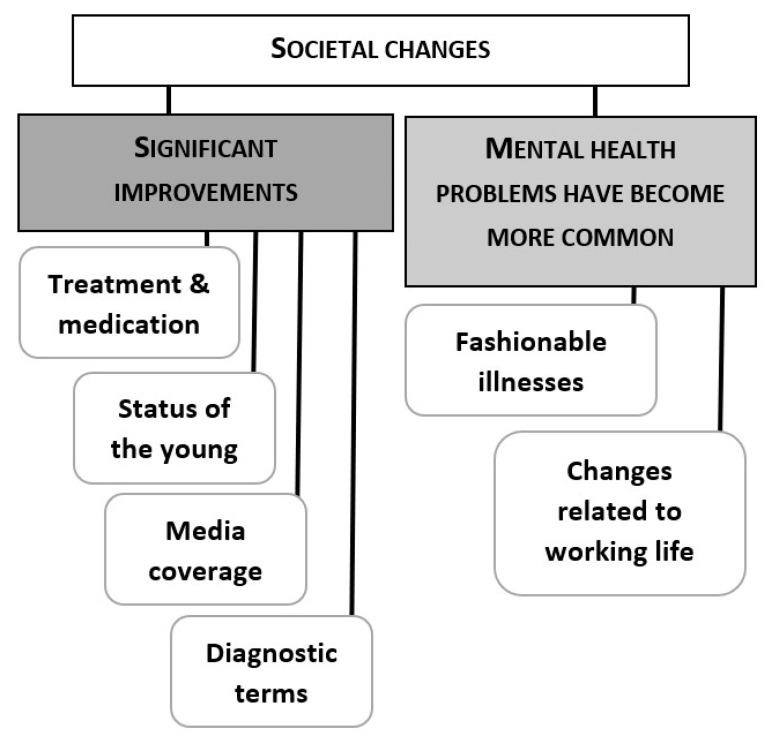
Societal changes that the focus groups perceived as elements that have reduced stigma.

**Figure 3:**
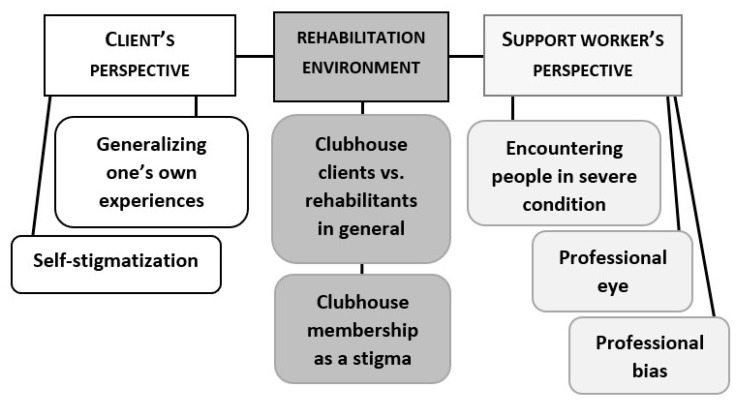
Factors related to perspective that are commonly used as reasons for believing in widespread stigmatization.

After the results of the national barometer were revealed, all of the 10 groups were positively surprised. When asked to contemplate the reasons for the divergence, the groups gave explanations that relied either on underestimating the effects of societal changes (Figure 2) or on having a different viewpoint compared to the informants in the national barometer (Figure 3). In particular, being a client or a staff member in mental health rehabilitation was often brought up as a reason for believing in widespread discrimination. We will analyze the groups’ reasoning in detail in the following two sections.

#### 4.2.1. Stigma and the Changing Society

In each group, there were participants who, after having heard the barometer result, reasoned that the situation with stigma must have improved significantly of late. However, the percentage of mental health rehabilitants that experience stigmatization because of their mental illness had in fact increased from the previous barometer results (47% in 2019, 39% in 2017, 39% in 2015) [44]. A closer analysis of the groups’ explanations reveals that the past, which was commonly acknowledged as “worse” with regard to stigma, is quite distant. The groups contrast involuntary long treatment periods in mental asylums with contemporary outpatient care and medication (Ext. 13–16). As a result, the attitudes toward mental illnesses are perceived to have improved linearly over time.

13. Venla, client: “There is a historical burden when you consider how the treatment has been. People were locked up in big institutions far from the city centers. The young have been born in a wholly different world.”
14. Jutta, client: “The hospitalization, as well, because I think that in the past, once you ended up in a mental hospital, you did not get out again.”
15. Jonna, client: “Could that have something to do with outpatient care being in (--) such good shape these days?”
16. Janette, support worker: “Medication has changed a lot during the time we have worked here. Like, one can actually see that people’s symptoms have changed and diminished, or that the drugs are more effective.”

The changes in diagnostic terminology were also perceived to increase acceptance of mental health rehabilitants. For example, changing “manic-depressive disorder” to “bipolar disorder” and “minimal brain dysfunction” to “ADHD” were mentioned as improvements that have reduced stigma. Another factor seen as separating the current times from the past in a positive way was publicity. Media attention and celebrities coming out with their mental health problems were seen as aspects that reduced stigma (Ext. 17, 18).

17. Netta, client: “Many celebrities have come out with having such problems, so ordinary people now also dare to become more public about it. Like, it isn’t a bad thing anymore but a strength.”
18. Janette, support worker: “This is wonderful news, and I think it could be due to the good work the celebrities have done here. Almost daily, one sees headlines and front pages that focus on mental health problems.”

Prior research by Ferrari [47] has indeed demonstrated that celebrities with mental health issues can be successfully used as a means to decrease the stigma connected to mental illness. However, as noted in previous research [5,6,48,49], the media also produce stigmatizing material about people with mental illness, connecting them to topics such as irrationality, violence, and threat. Perhaps because the groups were so focused on explaining “improvement” that only one of the groups discussed the negative effects of the media. They reminisced about past episodes of a Finnish soap opera and disapproved of its vision of mental illness.

Being open about mental health problems was regarded to be considerably easier for young people, as they have been accustomed to talking about mental health and have received information about it from an earlier age compared with previous generations (Ext. 19, 20). The participants thought it was easier for young people to talk about mental health with their peers and in public. Discussing and taking care of one’s health and wellbeing in general was seen as belonging to a culture accepted and maintained by the young (Ext. 21, see also Ext. 8 above). These examples show that the proactive denial strategies [50], which are used to challenge stigma, are above all connected to the young and the popular. However, the concept of who is “young” seems to vary; some of the participants who were in their early 30s contrasted themselves with young people.

19. Riina, client: “I told my grandma it is my illness. I tell whomever I want about it, but maybe it is because I am younger and I have grown into it and we have been taught about mental health.”
20. Tuula, client: “Maybe younger people understand these things better.”
21. Ida, support worker: “There is an age-related cultural difference. (--) it is in fashion, in quotation marks, to take care of one’s head. (--) overall, caring for mental health, in my opinion, is less of a taboo for young people.”

In two groups, some of the support workers connected the openness about mental health problems to a change in work ethics. They seem to suggest that talking about mental health problems and taking sick leave because of them are signs of a weak work ethic, although they represent these concepts as generalizations rather than their own personal views (Ext. 22, 23). Again, the young are regarded as different from the others; they pay attention to their mental health and risk being labeled as unreliable workers. There is indeed evidence of burnout sufferers struggling to legitimize being sick in a work-centered society such as Finland, because sick leave is often related to exploiting the welfare system [51]. The support workers in our interviews seem to place themselves somewhere between these two extremes. Things have changed in their lifetime, and they identify neither with the generation they were raised by nor the generation they regard as the youth of today.

22. Marko, support worker: “The older generation has strong work ethics, and this idea of sort of giving up psychologically does not fit in it. But, I mean, for a young person, it may be acceptable to admit that one does not manage or care.”
23. Support workers:
  Varpu: “I don’t know ANYONE my age who would have talked about having depression when we were young.”
  Maikki: “Nor do I.”
  Varpu: “Whereas nowadays it is completely.“
  Seija: “No. Back then people would go to work, and work until the end. For real, you definitely would not have taken a sick leave because of depression.”

However, other groups found a strong connection between changes in working life and the increase in mental health problems (Ext. 24, 25). They discussed how work has become more demanding, stressing, and fragmentary. Burnout was seen as a result of working life getting hard, not a change in work ethics among the young. One of the clients also proposed that the changes in working life explain why older people do not understand the young (Ext. 26).

24. Anja, support worker: “Many have gotten sick because of work.”
25. Venla, client: “They are so common, burnouts and everything. It can be so tough these days. I mean, like, you have to be in a very good condition to manage in the working life. It is a hard game nowadays.”
26. Kyösti, client: “Therefore, the older people don’t understand the younger ones because the working life has been so different.”

Nearly all of the groups reasoned that stigma may have diminished due to mental health diagnoses becoming more common. The idea behind this is that being familiar with mental illness decreases prejudice [52], and that those who have a diagnosis do not stand out as individuals as much as before. Several participants illustrated the situation by presenting figures they had learned somewhere (Ext. 27–29). One group of clients also found that the prevalence of mental health problems is linked to over-diagnosing (Ext. 29–30). Their impression was that nowadays, everyone can be diagnosed with something if examined closely enough. Therefore, the right to judge others has diminished. Some of them also claimed that people are classified as deviant without legitimate reasons (Ext. 30). This remark is in contrast with the notion of diminishing stigma. Classifying someone unnecessarily as deviant is, in essence, discrimination.

27. Jutta, client: “In Finland, it’s one in five, I suppose (mental health problems).”
28. Venla, client: “It really is a lot if nine people retire every day because of depression.”
29. Kale, client: “Nowadays, nobody stigmatizes anyone for anything because everyone is just as nuts there on the streets. Everyone should probably have some sort of a diagnosis but just haven’t been examined. (--). Forty years ago, only a couple of percent had ADHD, but now, pretty much the whole nation of Finland has it.”
30. Clients:
  Kyösti: “If you are overly well-mannered, that too is classified as being crazy these days.
  One shouldn’t be (--).”
  Kale: “It is abnormal.”
  Mari: “A good actor is a psychopath.”

Overall, all of the groups jointly and unanimously constructed a narrative of stigmatization diminishing over time, although they had underlined the persistence of stigma just a moment before in the evaluation task. Hence, the idea of having overestimated the prevalence seems to function as evidence of improvement. What is noteworthy is that despite discussing working life becoming harder and mental health problems, sick leave, and disability pensions becoming more and more common, none of the groups saw a possibility of negative development through which stigmatization would get stronger. The current issues were instead understood as attenuating factors that promote acknowledgement and inclusion.

The only departure from the narrative of progress with regard to stigmatization concerns the status of mental illnesses that the groups considered as serious conditions. Some participants claimed that the attitudes have improved only toward those whose conditions have recently become regarded as “fashionable”. Depression, burnout, ADHD, and bipolar disorder were named as popular conditions, whereas schizophrenia was regarded as unfavorable with respect to media appeal (Ext. 31).

31. Support workers:
  Taru: “It isn’t as media-sexy to feature a women’s magazine narrating a story about having paranoid schizophrenia. It is completely different from getting burnout.”
  Oona: “Burnout sounds like you are a hero and a victim. You have languished under a burden.”

Previous research has proven that social distance toward people with schizophrenia is larger than toward those with depression due to the anticipation of social disability [53]. People with schizophrenia also stand out in the unemployment and disability statistics, see, e.g., [54,55] and being unemployed is known to emphasize exclusion [51]. However, from the viewpoint of the Clubhouse organization, people with schizophrenia are a notable client group, and this may potentially reflect on the participants’ views of mental illness and stigma. Note, the Clubhouse organization does not keep registers of their clients’ diagnoses, but the communities promote themselves in schizophrenia-related contexts. In the next section, we will investigate how the participants themselves connected their conception of stigma to their position in the mental health scene.

#### 4.2.2. First-Hand Experience and the Rehabilitation Environment

When the groups were asked to contemplate the reasons for the divergence between their views and the barometer’s result, in addition to considering having underestimated how much society has changed, the groups underlined their own experiences and knowledge of the matter. A common interpretation was that mental health rehabilitation is an environment that enhances the awareness of stigma. Rehabilitation was perceived both as a context that supports a comprehensive understanding of stigma and as a context that distorts the perception. We will specify these views in the following.

Right from the first stage of the stimulation task, the client groups justified their high estimation of the prevalence of stigma with their first-hand experiences of prejudice and shame (see Section 4.1). In the later discussions, some of the clients contemplated that these experiences can easily be generalized (Ext. 32). In addition to their own experiences, the clients considered often hearing about incidents of stigma in the Clubhouse. They reckoned that frequent exposure to these narratives can potentially lead to overemphasizing them (Ext. 33).

32. Mari, client: “It depends on one’s own experience. It only takes a couple of babblers to make one draw a conclusion.”
33. Riina, client: “I think it stresses it, being here? Being with other mental health rehabilitants, one hears these stories about how people have been stigmatized more than one would in an average workplace. Maybe, in a way, that colors the world a bit darker.”

The support workers also acknowledged that the rehabilitation environment affects the way they perceive stigma (see Ext. 10–12 above). They saw a difference between the Clubhouse clients and rehabilitants in general (Ext. 34). They pondered the fact that, on average, the clients in the Clubhouse community have more serious mental illnesses than the informants in the national barometer, and that the social consequences are therefore also harder for the Clubhouse clients (Ext. 35). In addition, they reasoned that those who experience stigmatization the least are most likely to participate in surveys (Ext. 36). Although the logic is in line with research on psychiatric disorders and their social costs, cf., [53,54], there is also evidence that participating in the Clubhouse model decreases perceived stigma and promotes quality of life [56]. Therefore, the assumption that the informants in the national barometer do not represent the Clubhouse clients is somewhat surprising.

34. Varpu, support worker: “The sample matters, in a way. The sample we have here versus the sample in this (points at cards).”
35. Taru, support worker: “Maybe the reason why we think like this, I don’t know, could be that the people who come to the Clubhouse, the majority of them have serious (--) psychiatric illnesses? And that is no longer a little thing.”
36. Sari, support worker: “Those who experience stigma least and are active in general will also answer surveys.”

What is also remarkable is that both the support workers and the clients brought forward the notion that being a client of the Clubhouse can in itself be shameful and stigmatizing (Ext. 37–40). The support workers talked about clients who do not wish people outside the community to know about their membership and will, therefore, not feature in photographs or newspaper articles, or even greet staff members or other clients outside the house (Ext. 38, 39). Reportedly, some clients even refuse to sit near an open window in order to avoid exposure (Ext. 38). The fear of being connected to people with mental health problems is associated with the fear of being labeled as one, which is considered shameful or unjustified (Ext. 39: “They do not have anything. It is just the others who are totally ill”). Concealing their Clubhouse membership can thus be interpreted as a measure of self-protection (Ext. 40).

37. Kyösti, client: “There is probably a little stigma related to the Clubhouse as well, although it is nice to come here.”
38. Sari, support worker: “If you want to put up photos on Facebook or anywhere else, people do not want their faces there. Very few dare to give their names or want to give their names. It is always the same couple of people who give interviews about things with their real names. Then again, some don’t want to sit near an open window to avoid being seen from the outside. Or you see someone there on the street and they don’t want to greet you because everyone knows you work at the Clubhouse, and so on.”
39. Seija, support worker: “Some don’t want their pictures on the papers or anything else to avoid being connected to this kind of mental health place. They don’t dare walk with the group or say hello to me because someone might know that I work here and they could work it out. Many people we have here are completely healthy. They do not have anything. It is just the others who are totally (--) like, ill.”
  Veijo, client: “I don’t have the nerve to tell my neighbors that I visit a Clubhouse.”
  Kyösti, client: “No, no, no. Because it has a negative impact.”

These examples illustrate what Meisenbach [50] calls avoidance strategies: the stigma is accepted but not applied to one’s self. It is the others who have unwanted traits and associating with them is contagious (cf. courtesy stigma, [1,57,58,59,60]). What is noteworthy here is that the avoidance strategy is used outside the Clubhouse community in order to save face as a healthy person. In other words, although the Clubhouse clients actively seek the company of other mental health rehabilitants, they may also find it disagreeable with respect to their image. 

What differentiates the support workers’ accounts from the clients’ is that the support workers appeal not only to their experiences in the community but also to their professional viewpoint. Some of them reason that as professionals working in the mental health sector, they know how to evaluate the effects of mental health issues and, therefore, notice things that the rehabilitants themselves are blind to, particularly features related to a mental illness (Ext. 40, 41). By stating that the rehabilitants themselves are unable to recognize the features that are distracting to others, these support workers seem to imply that the stigma can “actually” be stronger than the experience reported by the rehabilitants in the barometer. Similarly, in the Finnish Mental Health Barometer, the majority (62%) of mental health professionals believed that people who experience problems with their mental health are stigmatized [44]. To some extent, the notion of a professional eye seems to reflect what Corrigan et al. [52] would classify as benevolent prejudice: the belief that people with mental illness are childlike and need to be monitored by compassionate caretakers (Ext. 40: Because we do watch them for a number of hours a day; 41: that perhaps makes us think “Oh dear”).

40. Hannele, support worker: “Because we do watch them for a number of hours a day and monitor their life situations and so on (--). Could it be that this rehabilitant experiences the thing differently or does not notice it, or that we, staff members, just see and feel it? Could it be something like this?”
41. Ida, support worker: “But per se, what you (Hannele) said earlier can be true. I don’t know how to put this now so that it sounds right, but, like, if a person does not necessarily (1.5) notice that much or internalize or recognize those features, that perhaps makes us think, *Oh dear*. A bit like that.”

Other support worker groups, however, discussed the possibility that the professional viewpoint can in fact cause bias and distort perceptions. First, meeting people day to day in certain circumstances, such as in the Clubhouse or in the hospital, may affect the image of the whole reference group (see Ext. 35 above). Second, the inner process of accepting one’s illness and regaining self-esteem as a “normal person” was acknowledged to be something that the professional viewpoint does not reach (Ext. 42). Not being able to reach the perspective of the ill is also reflected in Extract 43, in which support workers contemplate whether they “get offended” on the rehabilitants’ behalf. The same group also discussed the possibility that the professionals may actually be sustaining the prejudice by believing in it (Ext. 44).

42. Maikki, support worker: “Maybe the difference is in the knowledge. When you have an illness and you know about it and you know yourself, I mean, at that point when you accept it, you’d think that you are still the same. A normal person.”
43. Support workers:
  Veli: “Do we, then, get offended on their behalf?”
  Ulla: “Well, I guess so.”
  Henna: “Probably.”
44. Support workers:
  Henna: “Perhaps it gets emphasized at this job. Maybe. I don’t know.”
  Tanja: “But if WE think there is prejudice, then we MAINTAIN it.”
  Henna: “That’s right. Yes. We do.”
  Veli: “Mm, mm.”
  Tanja: “This is a very important question. This was enlightening.”

Of note, in the last extract, Tanja describes the interviewer’s question as “enlightening”: she has become conscious of something she was not aware of before. In fact, all of the groups found the stimulated interview beneficial in terms of introspection and peer support. We will return to this notion in the concluding section.

All in all, our study suggests that familiarity with mental health problems may not be as unilaterally connected to decreased prejudice as one may think based on previous research (such as [52]). The focus groups in our study consist of people who regularly socialize with mental health rehabilitants in an environment that campaigns against stigmatization (e.g., the Diagnosis Free Zone campaign). Yet, it seems that recurrent exposure to certain kinds of problems and narratives may in fact strengthen the belief in extensive discrimination, which, in turn, could possibly also confirm some marginalizing conceptions. 

## 5. Conclusions

In this study, we examined how professionals and clients of mental health rehabilitation units perceive, explain, and account for mental illness stigma as a phenomenon. We also compared the ways in which the specific accountabilities connected to the participants’ more specific roles became visible in their discussion. For the methodology, we used a stimulated focus group interview that was designed to orient the participants toward reasoning. The participants were first asked to evaluate the prevalence of stigma together and then to explain their viewpoint in detail. As the method allows thinking together and comparing and adjusting views within the group, it provided us with evidence about the societal understandings and opinions that the participants in a given group are ready to support in front of their reference group. The stimulation task was beneficial in the sense that it activated the discussion and provided a way to introduce the delicate topic in a similar fashion to each group.

All of the groups estimated the prevalence of experiences of stigma to be notably higher than the Finnish Mental Health Barometer (47%, see [44]). The support workers’ estimations were even higher than the clients’ own. Out of the three given options (47%, 62%, and 71%), all of the support worker groups decided on the highest percentage. A detailed analysis of the groups’ accounts revealed that all of the groups viewed stigma as a phenomenon that persists despite the efforts to reduce it. However, the clients’ perceptions were closely connected to their first-hand experiences of social losses and feelings of self-shame, whereas the support workers based their evaluations on what they had heard in the rehabilitation center, namely, narratives of diagnostic overshadowing in primary care, cf., [33]. Thus, the professionals viewed stigma as something that is inflicted upon the mentally ill, while the clients acknowledged stigma as a more complex phenomenon in which one’s own confidence and recovery also play a role. 

After hearing the national barometer’s result, all of the interviewed groups agreed that the level of stigma had decreased due to the development of society—unaware that the barometer rating had in fact increased. The groups talked about improvements in care and medicine, as well as the media’s role in raising mental health awareness. They also thought that stigma had diminished because mental health problems have become more common and believed that the younger generation would experience less marginalization than previous generations. The only departure from the narrative of progress with regard to stigmatization concerned the status of serious mental illnesses, such as schizophrenia. This is also reflected in the way the participants explained their particular perspective on the topic. They argued that the clients in the Clubhouse environment have more serious mental health issues than rehabilitants on the average, and that they, therefore, experience more marginalization. This idea is in line with what is known about the social costs of serious psychiatric disorders [53,54], but the view is in stark contrast to what the Clubhouse model has been designed for, namely, reducing stigma and supporting recovery [56]. 

There was also tension in the attitudes toward Clubhouse membership. Being a Clubhouse client was seen as empowering but also as stigmatizing with regard to the clients’ social status in general, which is why some of the clients keep their membership confidential. This shows that avoidance strategies [50] can be deployed even by clients of rehabilitation communities that promote inclusion and function as “diagnosis-free zones”. However, concealing one’s membership in order to protect one’s self from negative attention should perhaps not be interpreted only as a sign of shame but also as an expression of self-determination and agency. If the image of an average Clubhouse client is a seriously ill and marginalized individual, the desire to distinguish one’s self from it is not surprising. 

Although the support worker groups’ conceptions of stigma were more negative than those of the clients in terms of the prevalence and the rehabilitants’ role in the experience, the focus group discussions seemed to raise awareness on the limitations of their perspectives. While considering their high estimate of the prevalence of stigma, the groups identified several possible factors that might have caused bias in their views. They noticed that the majority of their clients have a serious mental health problem, and that this affects their image of the whole reference group. In addition, they posited that as professionals, they may monitor the signs of the illnesses from a different perspective than the clients. One of the groups even considered that they might be sustaining prejudice by believing in it. Thus, sharing and comparing thoughts on the sensitive matter with the support of an outsider seemed to enhance introspection. 

The study has certain limitations. Given that this study was qualitative, its aim was to capture in depth the concepts that were shared in the reference groups. The rehabilitation units were selected purposively so that they would be diverse, and the interviews were continued until the saturation point was reached and recurring themes were identifiable in the discussions. However, although the sampling method provided rich qualitative information, its results cannot be considered as representative of all Clubhouses. Neither can they be uncritically generalized to apply to professionals and mental health clients in other social and health care contexts and rehabilitation environments. As the participants themselves perceived that their conceptions were tightly linked to their rehabilitation environment, it can be assumed that studying a different type of rehabilitation environment could result in different outcomes and emphasis. It is also noteworthy that voluntary participation involves the bias of some people being less likely to participate than others. In the context of mental health rehabilitation, it can be assumed that the most marginalized rehabilitants are less likely to volunteer in studies like this. Nevertheless, some second-hand knowledge about the most vulnerable groups was attained from the participants who volunteered. 

In any case, our results pose a challenge to the idea that familiarity with mental illness decreases stigmatizing beliefs, cf., [2,52]. There is no doubt that the Clubhouse rehabilitation raises awareness of stigma and offers peer support and empowering experiences [37,38]. At the same time, however, the rehabilitation environment may also be enforcing some maligning beliefs and sustaining the perception of stigma as an inevitable reality in which the rehabilitants have no agency. As their experiences in the rehabilitation context shape their beliefs in various ways, it is important that mental health professionals regularly revisit their modes of operation and examine the possible biases in their perspective. 

With regard to serious mental illnesses, reducing stigma is particularly challenging, as the image of these illnesses seems to entail scarce possibilities of recovering and participating in society. While we do not have a solution to this well-known issue, our data inspired us to question whether it would be more fruitful to promote acceptance of diversity rather than issue instructions to avoid stereotypes, see, e.g., [2]. Respect should not rely upon a person’s position on the spectrum.
